# Microbiological substantiation of the effectiveness of quercitin and its combination with ethylmethylhydroxypyridine succinate in the complex treatment of odontogenic phlegmon and maxillofacial abscesses

**DOI:** 10.3389/froh.2024.1338258

**Published:** 2024-01-19

**Authors:** Kateryna Lokes, Anatolii Kiptilyi, Margaryta Skikevych, Dmytro Steblovskyi, Vitaliy Lychman, Serhii Bilokon, David Avetikov

**Affiliations:** ^1^ Department of Oral and Maxillofacial Surgery, Poltava State Medical University, Poltava, Ukraine; ^2^ Department of Pediatric Oral Surgery, Poltava State Medical University, Poltava, Ukraine

**Keywords:** odontogenic infections, quercitin, 2-ethyl-6-methyl-3-hydroxypyridine succinate, phlegmones, abscesses, maxillofacial area

## Abstract

Odontogenic infections can become life-threatening. The aim of the study was to determine the effectiveness of quercitin and its combination with ethylmethylhydroxypyridine succinate in the complex treatment of odontogenic infections by assessing the microbial population of the infection site.

**Materials and methods:**

The cross-sectional study included 75 patients. Purulent exudate was collected from the infection site. The number of colony-forming units was counted using the standard plate method.

**Results:**

The microbiological examination of purulent exudate obtained from the patients revealed the general prevalence of Gram-positive cocci. On the seventh day of treatment, the total number of microorganisms in the purulent exudate of patients in group I, whose treatment included a combination of the standard protocol with quercitin, significantly decreased compared to the first day of the same group. The results of treatment of patients in group II, which included the standard protocol in combination with quercitin and 2-ethyl-6-methyl-3-hydroxypyridine succinate, demonstrate a significant decrease in the total number of bacteria in the infection focus on the fifth day of treatment compared to this indicator of the group at the beginning of the study.

**Conclusions:**

When quercitin was used as part of complex treatment, the total treatment period was reduced by 1.4 days. However, the combined use of quercitin and ethylmethylhydroxypyridine succinate against the background of standard treatment of patients with odontogenic infection contributed to a reduction in hospital stay by 2 days.

## 1 Introduction

About 30.0% of patient visits for emergency surgical care are associated with odontogenic infection in highly developed countries, and in developing countries this figure is even higher (1, 2). Odontogenic diseases of maxillofacial localization are characterized by a rapid aggressive course against the background of a sharp deterioration in the patient's general condition with further spread of inflammation from one anatomical part to another. That is why today, despite the rapid development of surgical dentistry, the mortality rate associated with odontogenic infection in the world ranges from 10.0% (3, 4).

Odontogenic infections can become life-threatening if left untreated, and increase the physical costs of treatment for the patient and the financial costs for the health care system (1, 5). Therefore, the issue of improving the effectiveness of treatment of odontogenic diseases of the maxillofacial area by combining standard treatment protocols with new substances that have anti-inflammatory, antimicrobial, immunomodulatory or other properties is a priority for both scientists and practitioners.

For example, quercitin is a type of natural flavonoid, which has recently been widely used in medicine due to its rich spectrum of bioactive properties. The anti-apoptotic, anti-inflammatory, antioxidant and antibacterial effects of quercitin make it a potential substance for the development of new therapeutic combinations. That is why quercitin is used to treat such diseases as bone metabolic diseases, gastrointestinal diseases, cardiovascular, cerebrovascular diseases and dental infections. However, the quantitative evidence base for the use of quercitin is currently insufficient and it is more often used as a dietary supplement (6).

2-ethyl-6-methyl-3-hydroxypyridine is most often crystallized in the form of succinate to achieve antioxidant, nootropic, neuroprotective, anti-inflammatory and other pharmacological effects. Preliminary studies prove the role of ethylmethylhydroxypyridine succinate in enhancing the antibacterial action of some antibiotics. Therefore, it is interesting to study the use of 2-ethyl-6-methyl-3-hydroxypyridine succinate in combination with other substances to identify possible synergism (7).

It is known that odontogenic diseases have a bacterial genesis and develop as a result of the spread of pathogens in necrotized pulp in complicated caries, periodontal pockets in periodontitis or pericoronitis in case of difficult eruption of retained teeth into the underlying tissues (2, 4). It becomes obvious that, along with pathogenic representatives, the oral normobiota in such cases can acquire pathogenic properties and serve as a reservoir of infection (8). Given this fact, the microbial population of the infection focus is the target of etiotropic treatment and can act as markers of its effectiveness (9, 10).

Therefore, the aim of the study was to determine the effectiveness of quercitin and its combination with ethylmethylhydroxypyridine succinate in the complex treatment of odontogenic phlegmon and maxillofacial abscesses by assessing the microbial population of the infection site.

## 2 Materials and methods

### 2.1 Study population

The cross-sectional study included 75 patients who were treated for abscesses and phlegmon of the facial area in the Department of Maxillofacial Surgery of the Municipal Enterprise “M.V. Sklifosovsky Poltava Regional Clinical Hospital of the Poltava Regional Council” during 2023. The criteria for inclusion of patients in the study were: a clinical diagnosis of L02.00—facial skin abscess, L03.20—facial phlegmon according to ICD-10 of odontogenic genesis with the involvement of no more than two tissue spaces with the patient's personal consent to participate in the study. The exclusion criteria were: inconsistency of diagnosis L02.00 and L03.20 according to ICD-10, spread of the infectious process to three or more tissue spaces, diabetes mellitus, pregnancy, mental disorders, congenital and acquired immunodeficiencies, self-medication with antibiotics before admission to the hospital, and lack of desire to participate in the study.

Prior to the study, written informed consent was obtained from each participant after a detailed explanation of the purpose and protocols of the study, which were in line with the ethical principles of the Declaration of Helsinki on Ethical Principles for Medical Research Involving Human Subjects. The study was approved by the Biomedical Ethics Committee of PSMU (Minutes No.220, from 25.10.2023).

**Figure 1:**
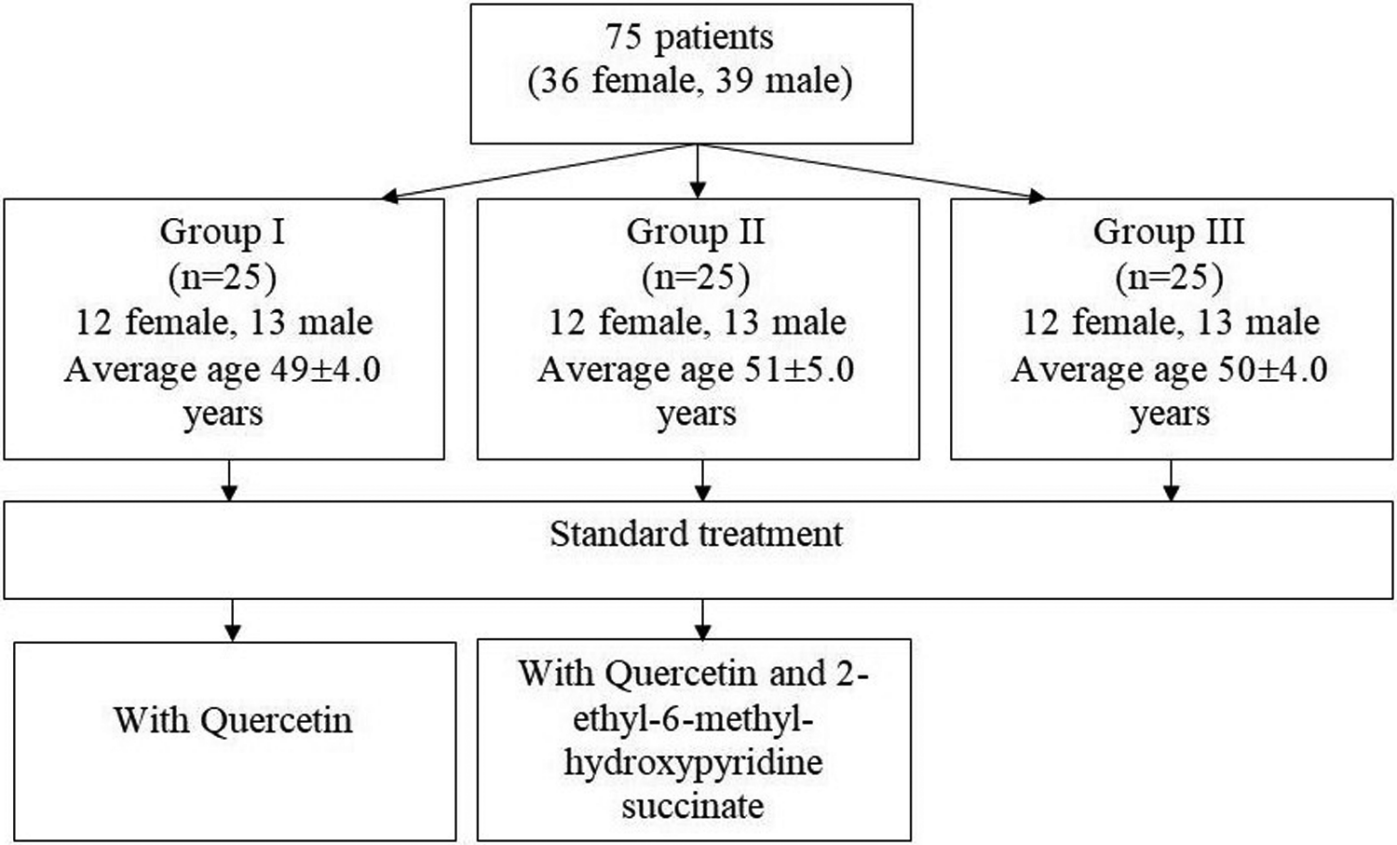
Flowchart of distribution of patients by groups.

Patients were empirically divided into three groups with an even distribution of patients by age and gender (Figure 1).

All patients (the first, second and third groups) underwent standard treatment according to the protocol, which included extraction of the causative tooth/teeth or roots, dissection of the infection site, followed by antiseptic treatment with 0.5% chlorhexidine and drainage with a corrugated rubber drain. Antibiotic therapy included Ceftriaxone 1.0 g 12-hourly and Metronidazole 100 mg daily. If it was necessary to replace the above antibacterial agents in case of allergy, the patient was automatically excluded from the study group. Pain control was performed with Ibuprofen 400 mg three times daily.

In patients included in the first group, conservative therapy included quercitin 1 g two times a day for 14 days (Quercitin, PJSC “Research and Production Center ‘Borshchahivskiy Chemical and Pharmaceutical Plant’”, Kyiv, Ukraine) additionally. The contents of a sachet with granules (2 g) were dissolved in 10 ml of water at about 50 degrees Celsius and infused for 15 min until homogeneous.

The standard treatment of patients in the second group included quercitin 1 g two times a day in combination with intravenous drip administration of 2-ethyl-6-methyl-3-hydroxypyridine succinate according to the regimen (Armadin, LLC SPC “Microkhim”, Rubizhne, Ukraine) additionally to the standard treatment:

1st day 50 mg 2 two times a day
2–3 days, 100 mg 2 two times a day
4th day, 100 mg 1 two times a day
5–6 days, 50 mg 1 two times a day

### 2.2 Microbiological studies

Purulent exudate was collected from the infection site using a sterile probe-tampon and placed in tubes with Amies medium for further transportation to the bacteriological laboratory of the Department of Microbiology, Virology and Immunology of PSMU within 24 h on the first, third, fifth, seventh, ninth, tenth and eleventh days of treatment. The samples were cultured on Columbia agar with 5% sheep blood (bioMarieux, France) and Thioglycolic medium (bioMarieux, France), at 37 °C under aerobic atmosphere for 24 h followed by culture isolation by standard culture method. The number of colony-forming units (CFUs) was counted using the standard plate method and expressed in decimal logarithms (lg). The conclusion about the etiological significance of the microorganism in the infectious-inflammatory process was made after isolation of microorganism in a monoculture or in the amount of 10^6^ CFUs or more. The final identification of isolates was carried out by morphological, tinctorial, cultural and biochemical characteristics using STROPTOtest 24, STAPHYtest 24 and ENTEROtest 16 (Erba, Czech Republic), according to the manufacturer's instructions.

### 2.3 Statistical analysis

The variational and statistical processing of the study results was performed using Microsoft Excel 2019 and 2022 GraphPad Software with the determination of the main variational indicators: mean values (M), standard errors (m), *p*-value (*p*). A one-way analysis of variance (ANOVA: one factor) was used to compare the results of the three groups of data. The Bonferroni correction adjusted the significance level to control for the overall probability of errors (false positives) for testing multiple hypotheses. The result was considered significant if the *p*-value was less than 0.05.

## 3 Results

The microbiological examination of purulent exudate obtained from the foci of odontogenic infection revealed the general prevalence of Gram-positive cocci (Table 1).

**Table 1:** Characterization of the qualitative composition of the microbiota of infection foci in phlegmon and abscesses of maxillofacial localization.

Microorganisms	Group І		Group ІI		Group ІII	
	Abs.	%	Abs.	%	Abs.	%
*S. aureus*	6	24.00	5	20.00	6	24.00
*S. auricularis*	ND	ND	1	4.00	ND	ND
*S. epidermidis*	3	12.00	2	8.00	2	8.00
*S. haemolyticus*	2	8.00	2	8.00	2	8.00
*S. saprophyticus*	ND	ND	1	4.00	ND	ND
*S. hominis*	2	8.00	1	4.00	2	8.00
*S. warneri*	1	4.00	ND	ND	ND	ND
*K. rosea*	1	4.00	1	4.00	1	4.00
*K. kristinae*	1	4.00	2	8.00	1	4.00
*S. pyogenes*	2	8.00	2	8.00	3	12.00
*E. faecalis*	4	16.00	3	12.00	3	12.00
*E. faecium*	3	12.00	4	16.00	4	16.00
*E. coli*	ND	ND	1	4.00	1	4.00
Total	25	100.00	25	100.00	25	100.00

Abs., absolute numbers of isolated species.

Representatives of the genus *Staphylococcus* were isolated on average from 50.67% of patients, 22.67% of which were *S. aureus* isolates. The most numerous species among coagulase-negative staphylococci (CoNS) were *Staphylococcus epidermidis* (*S. epidermidis*) and *Staphylococcus haemolyticus* (*S. haemolyticus*), with a frequency of 9.33% and 8.00%, respectively. Representatives of the genus *Enterococcus* were isolated in more than a quarter of patients (28.00%). Moreover, *E. faecalis* and *E. faecium* were found with almost equal frequency among patients of all three groups. It is worth noting that *Streptococcus* spp. as pathogens of maxillofacial abscesses and phlegmon were identified in only 7 patients, which was 9.33% of cases. A similar result was demonstrated by *K. rosea* and *K. kristinae*, the proportion of which was 9.33% in total. In turn, Gram-negative bacilli were isolated among the causative agents of abscesses and phlegmon of maxillofacial localization in only two patients, accounting for 2.67% of the total microbiota of the infection sites.

According to the results of the study, the total number of microorganisms colonizing the infection site in conditions of maxillofacial abscesses and phlegmon was on average 9.05 ± 0.50 log CFUs/ml. It is worth noting that the total microbial load in the three groups of patients on the day of admission to the hospital was at the same level and did not differ significantly (Table 2).

**Table 2:** Dynamics of microbial colonization of postoperative wounds in phlegmon and maxillofacial abscesses according to the type of treatment, CFUs/ml, log.

	Group І	Group ІI	Group ІII
1st day	9.04 ± 0.51	9.03 ± 0.51	9.08 ± 0.51
3rd day	8.46 ± 0.85	8.20 ± 0.85	8.78 ± 0.62
5th day	7.21 ± 0.89	3.82 ± 1.93^b^	7.73 ± 1.15
7th day	3.21 ± 0.54^a^	0.07 ± 0.34^b^	3.54 ± 1.12^c^
9th day	0	0	0.44 ± 1.12^c^
10th day	–	–	0.15 ± 0.50^c^
11th day	–	–	0

^a^ Reliability of the difference in the indicator relative to the indicator of the first day of Group I, *p* < 0.05.

^b^ Reliability of the difference in the indicator relative to the indicator of the first day of Group II, *p* < 0.05.

^c^ Reliability of the difference in the indicator relative to the indicator of the first day of Group III, *p *< 0.05.

Despite the fact that on the third and fifth days of treatment in patients of group I, the total microbial colonization of the infection sites decreased by 0.58 log and 1.83 log, respectively, the results did not have a significant difference from the baseline. It is worth noting that only on the seventh day of treatment, a significant decrease of 5.83 log in the total number of microorganisms in the purulent exudate of patients in group I, whose treatment included a combination of the standard protocol with quercitin, was observed compared to the first day of the same group (*р* < 0.05).

Analyzing the results of treatment of patients in group II, a slightly faster antimicrobial effect can be noted, as a significant decrease in the total number of bacteria by 5.21 log in the infection site was determined on the fifth day of treatment compared to this group at the beginning of the study (*р *< 0.05). However, there was one patient in this group with a severe course, who continued to isolate microorganisms in the amount of 1.70 log CFU/ml from the surgical wound on day 7 of treatment. Therefore, the complete eradication of microorganisms in patients in groups I and II was established on day 9 of the study.

In contrast, when treated according to the standard protocol, patients in group III showed a significant decrease in microbial colonization by 5.54 logs only on day seven, compared with the baseline of this group of patients (*р *< 0.05). However, on the ninth day, there were still 4 patients (16.0%), and on the tenth day, 2 (8.0%), whose purulent exudate was found to contain microorganisms. Therefore, complete eradication of postoperative wounds in patients of group III was recorded only on the eleventh day of treatment.

The data of microbiological studies correlated with the clinical data of objective examination of patients. Thus, a significant decrease in the number of microorganisms in the postoperative wound, compared with this indicator on the first day of treatment, coincided in time with a decrease or complete absence of purulent discharge, the appearance of granulations in the wound, and a decrease in collateral edema.

The study found that the average duration of treatment of maxillofacial abscesses and phlegmon according to the standard protocol was 8.4 ± 0.96 days. When quercitin was used as part of a complex treatment, this period was reduced by 1.4 days. However, the combined use of quercitin and ethylmethylhydroxypyridine succinate against the background of standard treatment of patients with odontogenic infection helped to reduce the length of stay in the hospital to an average of 6.1 ± 0.4 days.

## 4 Discussion

The frequency of isolation of staphylococci as a causative agent of inflammatory processes does not exceed 30%. About 15.0% of cases are isolates of *S. aureus* and the rest of them—coagulase-negative staphylococci. According to the literature, it is clear that Gram-positive cocci, including genera of *Staphylococcus*, *Streptococcus*, and *Enetrococcus*, prevail in the development of odontogenic infections of soft tissues (4, 11). The results obtained by us fully confirm this fact. After all, Gram-positive cocci have a wide arsenal of virulence factors, such as surface proteins that provide adhesion to epithelial cells, the ability to form biofilms and penetrate the bloodstream with subsequent spread throughout the body. All this contributes to the development of severe septic conditions (4, 8, 11).

The obtained results demonstrate the effectiveness of the standard protocol for the treatment of phlegmon and maxillofacial abscesses with the addition of quercitin or its combination with ethylmethylhydroxypyridine succinate.

In the last decade, a significant number of scientific studies have emerged indicating the use of flavanoids, including quercitin, in the treatment of metabolic bone diseases, gastrointestinal disorders, cardiovascular disorders, immunosuppression, and cerebrovascular diseases, including Alzheimer's disease (12–14). Preliminary *in vitro* studies prove the antimicrobial effect of different concentrations of quercitin on bacteria of different groups and fungi. Moreover, Gram-positive microorganisms showed the greatest sensitivity to this flavonoid (15, 16). Given the wide potential of quercitin bioactivity, the results of its use in the complex therapy of patients with phlegmon and maxillofacial abscesses were quite natural. After all, it has been previously proven that quercitin promotes the regeneration of hard and soft tissues of the human body by inhibiting the inflammatory mediator PGE2 and increasing the activity of alkaline phosphatase (17). In addition, this flavonoid suppresses the expression of cytokines and inducible nitric oxide synthase due to its ability to inhibit the NF-kappaB pathway without modifying the activity of c-Jun N-terminal kinase (18). We observed a significant decrease in the number of microorganisms in the infection site when quercitin was administered as part of the complex treatment of patients, which could be due to its ability to block the surface proteins of Gram-positive bacteria responsible for adhesion and biofilm formation (19, 20). Due to its antioxidant properties, anti-inflammatory, immunomodulatory, and antimicrobial effects, and the presence of an analgesic effect, quercitin promotes faster wound cleansing and healing and is quite promising for use in surgical dentistry (11).

It is known that 2-ethyl-6-methyl-3-hydroxypyridine succinate has anti-ischemic, antihypoxic, neuroprotective, anti-stress, nootropic, and geroprotective properties, so it is widely used in neurology, cardiology, and surgery (21, 22). Recently, there have been reports on the antibacterial effect of 2-ethyl-6-methyl-3-hydroxypyridine succinate, namely its ability to inhibit the growth of *Staphylococcus* spp., *Streptococcus* spp., *E. coli*, and yeast-like fungi of the genus *Candida* (22). Given the fact that among the microorganisms isolated by us from patients with phlegmon and maxillofacial abscesses, representatives of the genera *Staphylococcus* and *Streptococcus* prevailed, it is natural to reduce the overall microbial population of surgical wounds when using the drug as part of complex therapy (23). The authors are of the opinion that 2-ethyl-6-methyl-3-hydroxypyridine succinate has a bacteriostatic effect by inhibiting protein synthesis in the microbial cell, and also affects the structure and function of membranes and surface proteins (21).

In addition, 2-ethyl-6-methyl-3-hydroxypyridine succinate has been shown to have synergistic effects with traditional antimicrobials, enhancing their activity against microorganisms *in vitro* (21). Therefore, when it is combined with conventional treatment and quercitin, it is also possible to potentiate the effect of both the latter and the antibiotic used. However, this fact requires further investigation.

## 5 Conclusion

Gram-positive cocci, mainly *Staphylococcus* spp. and *Streptococcus* spp. play a dominant role in purulent exudate obtained from foci of odontogenic infection.

On the seventh day of treatment, the total number of microorganisms in the purulent exudate of patients in group I, whose treatment included a combination of the standard protocol with quercitin, significantly decreased by 5.83 log compared to the first day of the same group. The results of treatment of patients in group II, which included the standard protocol in combination with quercitin and 2-ethyl-6-methyl-3-hydroxypyridine succinate, demonstrate a significant decrease in the total number of bacteria by 5.21 log in the infection focus on the fifth day of treatment compared to this indicator of the group at the beginning of the study.

When quercitin was used as part of complex treatment, the total treatment period was reduced by 1.4 days. However, the combined use of quercitin and ethylmethylhydroxypyridine succinate against the background of standard treatment of patients with odontogenic infection contributed to a reduction in hospital stay to an average of 6.1 ± 0.4 days.

### 5.1 Limitations of the study

The study was conducted during the wartime, which may have affected the results due to the increased use of antibiotics and antiseptics.

The study only included bacteria from the patients of one hospital, which may limit the generalizability of the findings to other clinics.

The study only considered a limited number of antioxidants and did not explore the effectiveness of others.

## Data Availability

The raw data supporting the conclusions of this article will be made available by the authors, without undue reservation.
